# Global, Regional, and National Epidemiology of Pediatric Stroke, 1990–2021: A Systematic Analysis From the Global Burden of Disease Study

**DOI:** 10.1002/pdi3.70031

**Published:** 2025-12-31

**Authors:** Zhao Yang, Hanxiao Duan, Hao Chen, Hui Hu, Nandi Wang, Tiantian Luo, Jun Tang

**Affiliations:** ^1^ Department of Neurosurgery, Children's Hospital of Chongqing Medical University National Clinical Research Center for Child Health and Disorders Ministry of Education Key Laboratory of Child Development and Disorders Chongqing China; ^2^ Department of Neurobiology Army Medical University (Third Military Medical University) Chongqing China; ^3^ Chongqing Key Laboratory of Child Neurodevelopment and Cognitive Disorders Chongqing China

**Keywords:** epidemiology, ischemic and hemorrhagic subtypes, pediatric stroke, the global burden of disease study

## Abstract

Pediatric stroke, although relatively rare, poses considerable health risks with substantial morbidity and mortality. Despite its clinical impact, comprehensive global assessments of its long‐term trends and disparities remain limited. Using estimates from the Global Burden of Disease (GBD) 2021 study, we evaluated the burden of pediatric stroke—including both hemorrhagic stroke (HS) and ischemic stroke (IS)—across 204 countries and territories from 1990 to 2021. Our original analyses included the calculation of the estimated annual percentage change (EAPC) of age‐standardized rates, stratified by age, sex, and sociodemographic index (SDI). In 2021, there were approximately 2.7 million prevalent pediatric stroke cases worldwide, with HS contributing 41.4% and IS 58.6%. Globally, stroke‐related disability‐adjusted life years (DALYs) declined from about 5.9 million in 1990 to 2.4 million in 2021. However, the incidence among adolescents aged 10–19 years increased during this period. Marked geographic disparities were observed, with low‐SDI regions experiencing disproportionately higher burdens, particularly from HS. India recorded the highest number of DALYs and incident cases in 2021. These findings provide a comprehensive global analysis focused specifically on pediatric stroke, underscoring that although the overall burden has declined, persistent and widening disparities highlight the need for targeted strategies, improved early recognition and strengthened healthcare systems in resource‐limited regions.

## Graphical abstract

In 2021, the burden of pediatric stroke varied substantially across countries and regions. China and India had the highest prevalence, while India and Nigeria accounted for disproportionately high DALYs, indicating greater health loss per case. Marked geographic disparities underscore the need for region‐specific strategies.

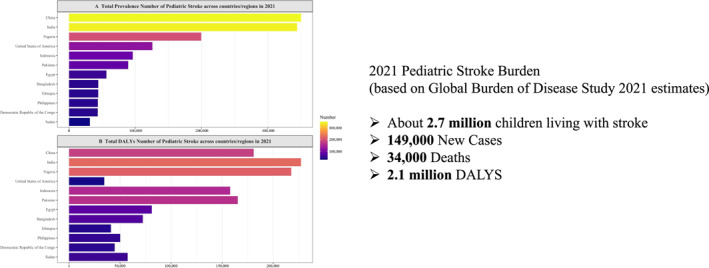

## 1 Introduction

Pediatric stroke, though relatively rare, is a devastating condition that significantly contributes to global childhood morbidity and mortality [1]. Classified as ischemic stroke (IS) or hemorrhagic stroke (HS), pediatric stroke often results in lifelong disability, severe cognitive impairments and substantial socioeconomic consequences for affected families and healthcare systems [2]. Despite its substantial impact, pediatric stroke remains under‐recognized and its epidemiology and risk factors are far less understood than those of adult stroke [3]. Prior Global Burden of Disease (GBD) studies have quantified the burden of stroke across all ages, but pediatric‐specific analyses remain limited. A recent GBD study also revealed that from 1990 to 2021, the incidence of stroke has continuously increased, whereas the pathogenic role of various risk factors has become increasingly significant. However, the study did not provide a detailed explanation or assessment of stroke in children [4]. To our knowledge, few investigations have systematically examined trends in pediatric stroke from 1990 to 2021 using stratified estimated annual percentage change (EAPC) analyses. Pediatric stroke frequently results in residual motor, language, or cognitive deficits and is associated with relatively high mortality rate (7.9%–25.3%) [5]. Early intervention can substantially improve functional outcomes, but infants and patients presenting with impaired consciousness tending to have poorer prognoses. However, current understanding of risk factors, epidemiology, and clinical outcomes remains inadequate.

Globally, the pediatric stroke burden has shifted over the past three decades. Advances in neonatal and pediatric care in high‐income countries have led to notable declines in mortality and disability [5]. However, the absolute burden remains considerable in low‐ and middle‐income countries (LMICs), largely driven by population growth and persistent healthcare disparities [6]. HS, including intracerebral and subarachnoid hemorrhage, accounts for the majority of pediatric stroke‐related disability‐adjusted life years (DALYs), particularly in resource‐limited settings where timely interventions are less accessible [7]. Conversely, IS, which is more prevalent in high‐income regions, has shown increasing incidence among older pediatric age groups [8].

The global distribution of pediatric stroke is further shaped by socioeconomic context. The sociodemographic index (SDI) has emerged as a critical determinant, with low‐SDI regions bearing disproportionately high burdens of both HS and IS. Regions, such as Sub‐Saharan Africa and South Asia, exhibit the highest prevalence and DALYs due to pediatric stroke, exacerbated by limited healthcare access and the prevalence of modifiable risk factors such as infections, congenital heart disease, and trauma [9].

Against this background, the present study aimed to provide a comprehensive assessment of the global, regional, and national burden of pediatric stroke between 1990 and 2021, using estimates from the GBD 2021 study. By evaluating trends in incidence, prevalence, mortality, and DALYs across 204 countries and territories—stratified by stroke subtype and SDI—we sought to identify key disparities and highlight opportunities for targeted interventions. Importantly, unlike previous GBD analyses that focused on stroke across all ages, our study specifically centers on the pediatric population and applies stratified EAPC analyses to quantify time trends and inequalities in stroke burden. This approach provides novel insights into the evolving global landscape of pediatric stroke and identifies priority regions for targeted prevention and resource allocation.

## 2 Methods

### 2.1 Study Design and Data Source

We retrieved estimates from the GBD 2021 results tool (Global Burden of Disease, Global Health Data Exchange) and the GBD Application Programming Interface (API). All indicators analyzed were model‐based estimates generated by the GBD consortium through systematic synthesis of multiple data sources using the DisMod‐MR 2.1 Bayesian meta‐regression framework. These estimates do not represent the directly observed case counts. In the GBD framework, stroke is defined according to the International Classification of Diseases (ICD) system, including codes I60–I69 (ICD‐10) and corresponding ICD‐9 codes. Subtypes were classified as IS (IS, I63), intracerebral hemorrhage (ICH, I61), subarachnoid hemorrhage (SAH, I60), and unspecified stroke (I64). HS was defined as the combination of ICH and SAH. Pediatric stroke was analyzed across eight GBD age groups: neonates (< 28 days), infants (1–5 months and 6–11 months), toddlers (12–23 months and 2–4 years), children (5–9 years), and adolescents (10–14 years and 15–19 years). All estimates were stratified by sex, age group, stroke subtype, and SDI quintile. The SDI is a composite indicator of development status, combining income per capita, average educational attainment, and fertility rates [10, 11].

### 2.2 Key Metrics

This study utilized modeled epidemiological estimates provided directly by the GBD 2021 study, including incidence, prevalence, deaths, and DALYs. These indicators represent outputs of GBD modeling rather than primary outcomes of this study. Accordingly, they were treated as inputs for secondary analyses performed herein. The study extracted, summarized, and compared these GBD‐derived estimates across age groups, sexes, regions, and SDI levels.

In addition to descriptive comparisons, we conducted secondary analyses to quantify temporal trends by calculating the EAPC based on GBD‐provided age‐standardized rates (ASRs). Thus, the EAPC serves as the primary analytical output of this study, capturing the magnitude and direction of long‐term changes in pediatric stroke burden between 1990 and 2021. In this study, all age‐standardized rates refer specifically to the pediatric population (aged 0–19 years), unless otherwise specified.

Definitions of key indicators are provided below to ensure conceptual clarity and consistency with the GBD framework:

Incidence/Incidence rates: Incidence was defined as the number of new cases occurring within a specified period. Incidence rates is expressed as the number of new cases per 100,000 person‐years.

Prevalence/Prevalence rates: In the GBD framework, prevalence refers to the number of children and adolescents living with the sequelae or long‐term consequences of stroke, including both recent and historical cases. Because stroke is an acute event, this measure does not indicate active acute episodes but rather individuals who have survived a prior stroke and remain alive with residual neurological or functional impairments. Prevalence rate is the proportion of a population living with a specific disease at a given time, typically expressed per 100,000 population.

Deaths: Total number of pediatric stroke‐related deaths, reflecting the direct mortality burden.

DALYs: A composite indicator integrating years of life lost (YLLs) due to premature mortality and years lived with disability (YLDs) because of stroke as estimated by the GBD modeling framework.

EAPC: Estimated annual percentage change, a measure of the temporal trend in age‐standardized rates over time.

All metrics were extracted directly from the GBD 2021 study, ensuring comparability with previously published global analyses.

## 3 Statistical Analysis

Incidence, prevalence, deaths, and DALYs were taken as modeled estimates provided by the GBD 2021 study and used as inputs for the secondary analyses described below.

ASRs reported by the GBD 2021 study were used for cross‐population comparisons to adjust for differences in demographic structures. No recalculation of ASR was performed by the authors; all ASR values were obtained directly from the GBD database. To evaluate temporal trends, we calculated the EAPC using a log‐linear regression model based on the GBD‐provided ASRs as follows:

$$\ln\left(ASR_{y}\right)=\alpha +\beta \times {\mathrm{year}}_{y}+\varepsilon_{y}$$

In this equation, $ASR_{y}$ represents the GBD‐reported age‐standardized rate in year y, β is the regression slope, and $\varepsilon_{y}$ is the random error term. The EAPC and its 95% uncertainty interval (UI) were computed as follows: $EAPC=100\times \left(e^{\beta }-1\right)$

$$\mathrm{Lower}=100\times \left(e^{\beta -1.96\times SE}-1\right),\mathrm{Upper}=100\times \left(e^{\beta +1.96\times SE}-1\right)$$

In this equation, SE is the standard error of β.

All analyses were performed using R Studio (version 1.4.110). Graphs were generated with R and GraphPad Prism (version 8.0). Because this study relied solely on publicly available de‐identified GBD estimates, ethical approval was not required. The study was not prospectively registered on an open research platform, which is acknowledged as a limitation.

## 4 Results

### 4.1 Global Trends in Pediatric Stroke (1990–2021)

**FIGURE 1:**
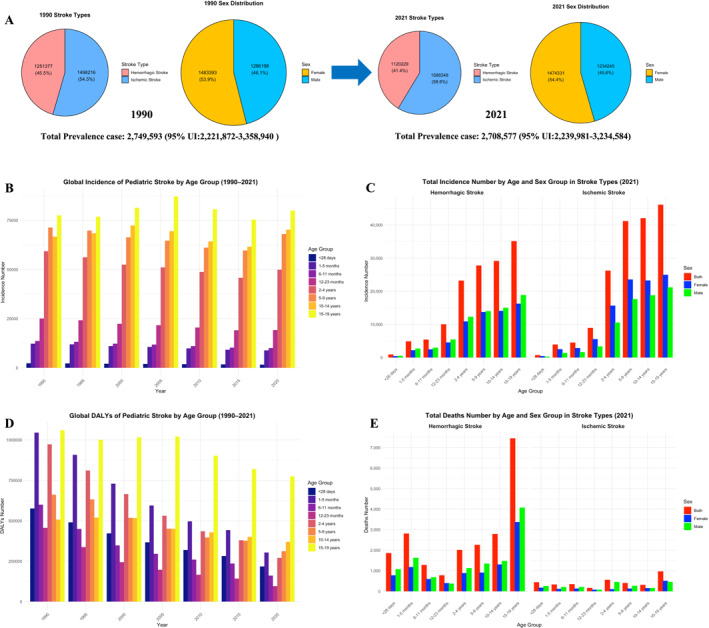
Global trends and demographics of pediatric stroke (1990–2021).

In 2021, there were an estimated about 2.7 million prevalent cases of pediatric stroke worldwide (95% UI: 2.24–3.23 million), comprising 41.4% HS and 58.6% IS, with females accounting for 54.4% and males for 45.6% (Figure 1A). This represents a slight decline compared with 1990 (about 2.75 million; 95% UI: 2.22–3.36 million). The number of incident cases among adolescents aged 10–19 years increased from approximately 144,300 (95% UI: 71,000–251,400) in 1990 to 152,500 (95% UI: 75,600–265,100) in 2021. Across age groups, incident cases rose with increasing age (Figure 1B). For example, in 2021, neonates (< 28 days) experienced about 1600 incident cases (95% UI: 1100–2400), whereas adolescents aged 15–19 years had 81,300 incident cases (95% UI: 42,000–136,300) (Table 1). The incidence of both HS and IS increased progressively with age, peaking in adolescents aged 15–19 years. Across all age groups, males exhibited higher HS incidence, whereas females accounted for a greater proportion of IS cases (Figure 1C). By stroke type, IS incidence exceeded HS incidence (IS: about 173,500 cases; 95% UI: 78,300–320,600 vs HS: about 136,400; 95% UI: 80,300–217,500). Notably, the ASIR for IS showed slight increases in those aged 10–14 years (EAPC = 0.51; 95% UI: −4.20–5.64) and 15–19 years (EAPC = 0.50; 95% UI: −3.66–4.89) (Table 1). Sex differences were also observed: males had a higher burden of HS (about 71,900 cases; 95% UI: 43,000–114,500), whereas females were more affected by IS (about 98,800 cases; 95% UI: 44,800–181,100) (Table S1).

**TABLE 1:** Number of incidence, DALYs, and deaths of stroke stratified by stroke type and age group in the pediatric population, and estimated annual percent changes from 1990 to 2021.

Age group	Absolute number (1990, 95% UI)			Absolute number (2021, 95% UI)			1990–2021 EAPC (95% UI)		
	Incidence	DALYs	Deaths	Incidence	DALYs	Deaths	Incidence	DALYs	Deaths
Pediatric stroke									
< 28 days	2,300 (1,500–3,400)	575,600 (431,600–788,200)	6,400 (4,800–8,800)	1,600 (1,100–2,400)	207,100 (152,900–277,000)	2,300 (1,700–3,100)	−1.13 (−3.62, 1.50)	−3.24 (−5.15, −1.42)	−3.53 (−3.83, −3.22)
1–5 months	12,300 (8,300–18,000)	1,045,200 (753,900–1,511,900)	11,600 (8,400–16,800)	8,800 (5,900–13,100)	282,700 (212,400–364,200)	3,100 (2,400–4,100)	−1.08 (−3.55, 1.46)	−4.13 (−6.14, −2.32)	−1.64 (−1.89, −1.39)
6–11 months	13,700 (9,400–19,600)	599,200 (390,800–981,200)	6,700 (4,400–11,000)	10,000 (6,900–14,500)	146,900 (89,500–227,200)	1,600 (1,000–2,500)	−1.02 (−3.34, 1.39)	−4.43 (−7.44, 1.73)	−2.43 (−2.70, −2.16)
12–23 months	25,100 (17,000–35,700)	456,300 (323,300–706,200)	5,100 (3,600–7,900)	18,900 (12,900–27,400)	87,300 (66,000–115,400)	900 (700–1,300)	−0.90 (−3.24, 1.55)	−5.19 (−7.36, −3.27)	−2.63 (−2.79, −2.47)
2–4 years	59,300 (35,200–101,600)	972,200 (637,600–1,642,400)	10,900 (7,000–18,500)	49,400 (28,200–87,600)	247,700 (146,300–399,500)	2,600 (1,400–4,300)	−0.58 (−4.05, 2.99)	−4.31 (−7.50, −1.50)	−2.12 (−2.24, −2.00)
5–9 years	71,300 (30,600–130,700)	661,000 (526,800–858,200)	6,900 (5,400–9,200)	69,000 (28,200–127,900)	301,600 (254,700–354,000)	2,700 (2,200–3,200)	−0.11 (−4.83, 4.72)	−2.50 (−3.84, −1.27)	−3.17 (−3.25, −3.09)
10–14 years	66,800 (31,000–120,100)	507,500 (423,700–613,900)	4,900 (4,100–6,000)	71,200 (33,600–128,800)	364,900 (305,900–430,500)	3,100 (2,600–3,600)	0.21 (−4.02, 4.70)	−1.06 (−2.22, 0.05)	−1.96 (−2.05, −1.87)
15–19 years	77,500 (40,000–131,300)	1,059,700 (923,900–1,193,900)	12,200 (10,500–13,700)	81,300 (42,000–136,300)	776,400 (663,100–905,200)	8,400 (7,200–9,800)	0.15 (−3.61, 4.04)	−1.00 (−1.88, −0.07)	−1.78 (−1.85, −1.72)
Hemorrhagic stroke									
< 28 days	1,400 (1,000–1,900)	504,600 (377,000–689,000)	5,600 (4,200–7,700)	900 (700–1,200)	167,700 (124,100–222,200)	1,900 (1,400–2,500)	−1.42 (−3.45, 0.74)	−3.49 (−5.38, −1.69)	−3.77 (−4.07, −3.47)
1–5 months	7,500 (5,300–10,300)	958,900 (691,900–1,380,500)	10,700 (7,700–15,400)	4,900 (3,500–6,600)	252,800 (190,200–325,700)	2,800 (2,100–3,600)	−1.38 (−3.41, 0.72)	−4.21 (−6.19, −2.40)	−1.80 (−2.05, −1.55)
6–11 months	8,200 (5,900–11,000)	497,000 (326,800–793,700)	5,600 (3,600–8,900)	5,400 (4,000–7,300)	115,400 (69,900–177,400)	1,300 (800–2,000)	−1.32 (−3.24, 0.68)	−4.60 (−7.54, −1.95)	−2.65 (−2.93, −2.37)
12–23 months	14,600 (10,400–19,500)	398,500 (281,600–614,000)	4,500 (3,200–6,900)	10,000 (7,200–13,400)	70,700 (53,100–93,700)	800 (600–1,000)	−1.22 (−3.14, 0.81)	−5.42 (−7.59, −3.49)	−3.01 (−3.19, −2.84)
2–4 years	31,000 (21,200–48,200)	847,700 (551,900–1,439,600)	9,600 (6,200–16,400)	23,200 (15,500–36,500)	185,900 (110,300–302,200)	2,000 (1,100–3,400)	−0.93 (−3.59, 1.77)	−4.78 (−7.95, −1.92)	−2.47 (−2.59, −2.35)
5–9 years	32,100 (16,300–54,200)	556,700 (442,700–727,400)	6,200 (4,800–8,300)	27,800 (13,700–47,500)	222,000 (190,300–256,600)	2,300 (1,900–2,700)	−0.47 (−4.33, 3.50)	−2.92 (−4.23, −1.74)	−3.40 (−3.48, −3.32)
10–14 years	38,000 (21,800–61,800)	407,000 (345,800–486,000)	4,500 (3,800–5,500)	29,100 (15,600–48,700)	267,300 (231,800–306,700)	2,800 (2,400–3,300)	−0.18 (−3.79, 3.57)	−1.35 (−2.36, −0.39)	−2.14 (−2.23, −2.05)
15–19 years	76,100 (43,400–123,300)	880,000 (776,300–978,700)	11,000 (9,500–12,400)	35,100 (20,100–56,300)	607,800 (525,400–702,000)	7,500 (6,400–8,700)	−0.26 (−3.56, 3.11)	−1.19 (−1.99, −0.32)	−1.85 (−1.92, −1.78)
Ischemic stroke									
< 28 days	900 (500–1,400)	71,000 (54,600–99,200)	800 (600–1,100)	700 (400–1,200)	39,400 (28,800–54,800)	400 (300–600)	−0.71 (−3.87, 2.55)	−1.88 (−3.91, 0.01)	−2.99 (−3.28, −2.70)
1–5 months	4,800 (3,000–7,800)	86,300 (62,000–131,400)	1,000 (700–1,500)	3,900 (2,400–6,400)	29,900 (22,200–38,500)	300 (200–400)	−0.66 (−3.75, 2.47)	−3.36 (−5.57, −1.53)	−1.67 (−1.92, −1.43)
6–11 months	5,400 (3,600–8,700)	102,200 (64,000–187,500)	1,100 (700–2,100)	4,500 (2,900–7,200)	31,500 (19,500–49,800)	300 (200–600)	−0.60 (−3.46, 2.31)	−3.72 (−7.04, −0.80)	−2.46 (−2.71, −2.21)
12–23 months	10,400 (6,600–16,200)	57,800 (41,700–92,100)	600 (400–1,000)	8,900 (5,600–14,100)	16,600 (12,900–21,700)	200 (100–200)	−0.50 (−3.36, 2.45)	−3.94 (−6.14, −2.08)	−2.16 (−2.31, −2.00)
2–4 years	28,200 (14,000–53,400)	124,500 (85,600–202,800)	1,300 (800–2,100)	26,200 (12,700–51,100)	61,800 (36,000–97,200)	600 (300–1,000)	−0.24 (−4.52, 4.27)	−2.24 (−5.43, 0.41)	−1.56 (−1.69, −1.43)
5–9 years	39,200 (14,200–76,500)	104,300 (84,200–130,700)	700 (500–1,000)	41,200 (14,400–80,400)	79,600 (64,400–97,400)	400 (300–500)	0.16 (−5.24, 5.74)	−0.87 (−2.26, 0.47)	−2.50 (−2.59, −2.40)
10–14 years	35,900 (14,600–68,200)	100,500 (77,900–127,900)	400 (300–500)	42,000 (18,000–80,100)	97,600 (74,100–123,800)	300 (300–400)	0.51 (−4.20, 5.64)	−0.10 (−1.75, 1.51)	−1.68 (−1.80, −1.56)
15–19 years	39,500 (18,200–69,500)	179,700 (147,600–215,200)	1,100 (1,000–1,300)	46,100 (21,900–80,100)	168,600 (137,700–203,200)	1,000 (800–1,100)	0.50 (−3.66, 4.89)	−0.21 (−1.43, 1.04)	−1.33 (−1.45, −1.21)

*Note:* Incidence, the number of new cases occurring within a specified period; Numbers are rounded to the nearest hundred.

Abbreviations: DALYs, Disability‐adjusted life years; EAPC, estimated annual percent change.

From 1990 to 2021, the number of deaths and DALYs attributable to pediatric stroke declined across all age groups, although the overall burden remains substantial. In 2021, there were about 2.41 million DALYs (95% UI: 1.89–3.07 million) and about 24,700 death (95% UI: 11920–31900) globally (Figure 1D,E). HS accounted for 78.3% of DALYs and 85.9% of deaths (Table S1). The highest burden was observed among adolescents aged 15–19 years, with about 776,400 DALYs (95%UI: 663,100–905,200) and about 8400 deaths (95% UI: 7200–9800) (Table 1). By stroke type, HS contributed disproportionately to disease burden, with about 1.89 million DALYs (95% UI: 1.50–2.39 million) and about 21,400 deaths (95% UI: 16,700–27,100), compared with IS, which caused about 525,000 DALYs (95% UI: 395,600–686,400) and about 3500 deaths (95% UI: 2600–4800) (Table S1).

Overall, although the pediatric stroke burden has declined substantially over the past 3 decades, specific increases in IS incidence among older children and adolescents, coupled with the persistently high DALYs and mortality associated with HS, highlight the need for continued targeted interventions.

### 4.2 SDI‐Based Trends

In 2021, the highest number of prevalent pediatric stroke cases was observed in middle‐SDI regions, with approximately 764,000 cases (95% UI: 618,900–929,000), representing a significant decline compared with 1990 (EAPC: −0.78; 95% UI: −0.83 to −0.74) (Table 2). By contrast, low‐SDI and low‐middle‐SDI regions exhibited the fastest increasing trends in prevalent cases, as indicated by the highest EAPCs (3.12 and 0.84, respectively). Within low‐SDI regions, both HS‐ and IS‐related prevalent cases rose across all age groups. For example, HS‐related prevalent cases increased from about 146,600 (95% UI: 123,200–173,600) in 1990 to about 267,200 (95% UI: 227,000–313,700) in 2021, whereas adolescents aged 15–19 years experienced an increase from about 48,000 cases (95% UI: 40,100–56,500) to about 96,000 cases (95% UI: 81,400–112,400).

**TABLE 2:** Number of prevalence, incidence and DALYs of pediatric stroke stratified by stroke type and SDI level, 1990–2021.

Stroke type	High SDI (1990/2021, [EAPC])	High‐middle SDI (1990/2021, [EAPC])	Middle SDI (1990/2021, [EAPC])	Low‐middle SDI (1990/2021, [EAPC])	Low SDI (1990/2021, [EAPC])
Pediatric stroke					
Prevalence	330,210/270,014 (−0.59)	510,629/308,004 (−1.28)	1,007,382/764,029 (−0.78)	572,522/722,401 (0.84)	32,402/641,887 (3.12)
Incidence	31,475/23,099 (−0.86)	57,180/33,748 (−1.32)	118,372/87,192 (−0.85)	75,495/86,470 (0.47)	45,496/79,375 (2.40)
DALYs	221,290/76,835 (−2.11)	763,059/156,186 (−2.57)	1,811,397/536,944 (−2.27)	2,056,552/796,580 (−1.98)	1,019,255/845,201 (−0.55)
Hemorrhagic stroke					
Prevalence	127,828/99,062 (−0.73)	235,790/127,961 (−1.48)	479,821/324,771 (−1.04)	260,181/300,268 (0.50)	146,591/267,168 (2.65)
Incidence	13,012/9059 (−0.98)	28,915/14,522 (−1.61)	61,294/38,960 (−1.18)	37,584/38,019 (0.04)	22,893/35,826 (1.82)
DALYs	159,642/46,916 (−2.28)	644,792/110,615 (−2.67)	1,559,944/416,021 (−2.37)	1,795,307/636,646 (−2.08)	886,280/677,087 (−0.76)
Ischemic stroke					
Prevalence	202,381/170,952 (−0.50)	274,839/180,043 (−1.11)	527,560/ 439,258 (−0.54)	312,341/422,133 (1.13)	179,810/374,720 (3.50)
Incidence	18,463/14,040 (−0.77)	28,265/19,226 (−1.03)	57,077/48,232 (−0.50)	37,910/48,450 (0.90)	22,603/43,550 (2.99)
DALYs	61,648/29,920 (−1.66)	118,267/45,571 (−1.98)	251,453/120,923 (−1.67)	261,245/159,935 (−1.25)	132,975/168,114 (0.85)

*Note:* Prevalence, the number of children and adolescents living with long‐term neurological or functional sequelae after stroke; incidence, the number of new cases occurring within a specified period; Countries were categorized into five SDI groups (low, low‐middle, middle, high‐middle and high SDI) according to their SDI levels in 2021, as defined by the GBD 2021 study. Numbers are rounded to the nearest hundred.

Abbreviations: DALYs, disability‐adjusted life years; EAPC, estimated annual percentage change; SDI, sociodemographic index.

**FIGURE 2:**
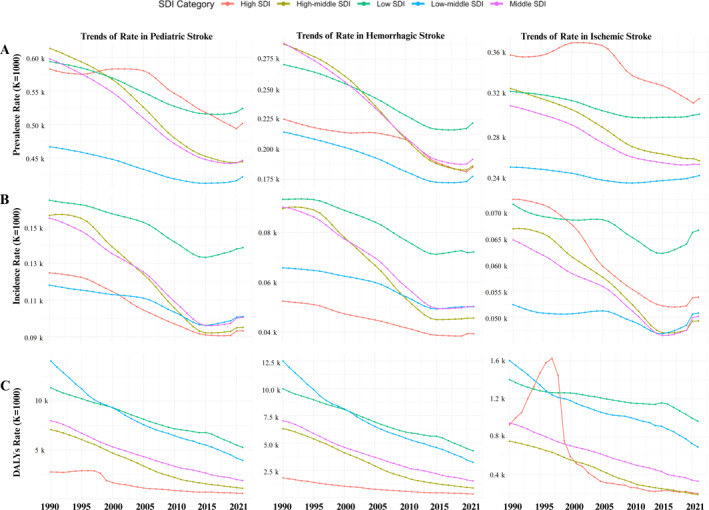
Temporal trends in pediatric, hemorrhagic, and ischemic stroke rates (1990–2021). ASDR, age‐standardized DALYs rate; ASIR, age‐standardized incidence rate; ASPR, age‐standardized prevalance rate; DALYs, disability‐adjusted life years; SDI, sociodemographic index.

Age‐standardized prevalence rate (ASPR) decreased overall from 1990 but showed a modest rebound from 2015 to 2021. In 2021, the highest ASPR of HS was observed in low‐SDI regions (222.0 per 100,000; 95% UI: 211.0–233.1), while the highest ASPR of IS was reported in high‐SDI regions (316.3 per 100,000; 95% UI: 285.3–347.3) (Figure 2A). Similar patterns were observed in incidence, with ASIR increasing slightly after 2015 across all SDI levels. The highest ASIR of HS and IS in 2021 occurred in low‐SDI regions, at 72.1 per 100,000 (95% UI: 65.5–78.7) and 66.6 per 100,000 (95% UI: 53.8–79.5), respectively (Figure 2B).

In terms of DALYs, substantial reductions were seen across all age groups in high‐, high‐middle‐, and middle‐SDI regions between 1990 and 2021 (Table 2). However, in low‐SDI regions, adolescents aged 10–14 and 15–19 years experienced rising DALYs burden for both HS and IS. Similar increases in IS‐related DALYs were also observed in low‐middle‐SDI regions among adolescents aged 15–19 years, consistent contrast with global trends.

Age‐standardized DALY rates (ASDRs) declined globally from 1990 to 2021, but the highest ASDR in 2021 was concentrated in low‐SDI regions, with HS‐related DALY rates of 4157.3 per 100,000 (95% UI: 2742.2–6120.3) and IS‐related DALY rates of 937.4 per 100,000 (95% UI: 633.6–1347.2) (Figure 2C).

### 4.3 Disparities by SDI

Across the 21 geographic regions, the highest number of prevalent pediatric stroke cases in 2021 was reported in South Asia (about 485,700; 95% UI: 381,600–606,700), whereas the lowest was observed in Australasia (about 5200; 95% UI: 4300–6200) (Table S2). Eight regions demonstrated increases in prevalent cases over the study period, including all Sub‐Saharan African regions, Australasia, North Africa and the Middle East, Oceania, and South Asia, with the most pronounced rise in Western Sub‐Saharan Africa (EAPC: 2.60; 95% UI: 2.56–2.64). Both HS‐ and IS‐related prevalent cases increased markedly in this region, with EAPCs of 2.35 (95% UI: 2.30–2.38) and 2.81 (95% UI: 2.77–2.86), respectively. Preschool‐aged children carried the heaviest burden in Western Sub‐Saharan Africa (about 46,500 cases; 95% UI: 42,000–51,600; EAPC: 2.08; 95% UI: 2.04–2.09), whereas South Asia exhibited the highest number of cases in post‐school‐age groups (about 460,200; 95% UI: 360,900–575,600; EAPC: 0.76; 95% UI: 0.69–0.84).

**FIGURE 3:**
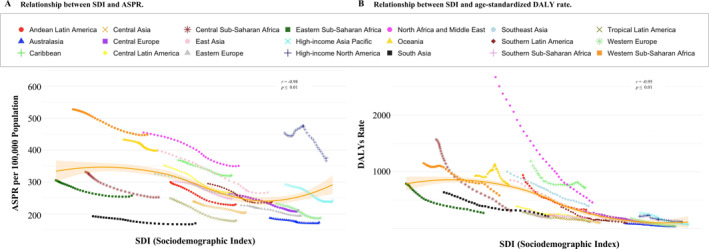
Geographic and sociodemographic trends in pediatric stroke burden (2021).

A strong inverse association was observed between SDI and pediatric stroke burden (Figure 3A). Regions with lower SDI, such as Western Sub‐Saharan Africa (SDI 0.45) and Oceania (SDI 0.47), exhibited the highest ASPRs, reflecting persistent gaps in healthcare access and a high prevalence of modifiable risk factors including infection and trauma. Conversely, East Asia (SDI 0.73) recorded the greatest decline in prevalence burden (EAPC: −1.28; 95% UI: −1.31 to −1.22), particularly among preschool‐aged children (EAPC: −1.63; 95% UI: −1.66 to −1.58), likely reflecting improvements in neonatal care and risk factor management.

In 2021, Western Sub‐Saharan Africa also reported the highest number of DALYs associated with pediatric stroke (about 506,000; 95% UI: 289,700–784,100), increasing over time (EAPC: 1.17; 95% UI: 0.87–1.46), whereas high‐income Asia Pacific experienced the largest decline (about 9300 DALYs; 95% UI: 7300–11,300; EAPC: −5.08; 95% UI: −5.19 to −5.08). In Western Sub‐Saharan Africa, HS contributed nearly three times as many DALYs (about 377,800; 95% UI: 214,300–594,000) compared with IS (about 128,200; 95% UI: 75,500–190,100). Preschool‐aged children represented the heaviest DALY burden in Western Sub‐Saharan Africa (about 302,900; 95% UI: 145,600–515,800), whereas postschool‐aged individuals were most affected in South Asia (about 277,500; 95% UI: 217,400–347,400). Globally, East Asia recorded the sharpest reduction in DALY rates (EAPC: −5.55; 95% UI: −7.08 to −3.91), followed by North Africa and the Middle East (EAPC: −5.49; 95% UI: −6.03 to −5.29). In 2021, six regions (Caribbean, Central Sub‐Saharan Africa, North Africa and Middle East, Oceania, Southeast Asia and Western Sub‐Saharan Africa) reported DALY rates higher than the global mean (280 per 100,000), whereas the rest 15 regions reported lower rates (Table S2).

### 4.4 National and Territory Trends in Pediatric Stroke

Among 204 countries and territories, China had the highest number of prevalent pediatric stroke cases in 2021 (about 350,200; 95% UI: 275,900–439,200), followed closely by India (about 344,200; 95% UI: 265,400–434,700). By subtype, China reported the highest number of HS cases (about 146,800; 95% UI: 115,400–184,500), whereas India recorded the highest number of IS cases (about 203,500; 95% UI: 157,400–256,800). Across all countries and territories, the mean number of IS cases (about 7800; 95% UI: 6400–9300) exceeded that of HS (about 5500; 95% UI: 4500–6700) (Table S3).

At the national level, Nauru recorded the highest ASPR of pediatric stroke (about 1700 per 100,000; 95% UI: 1600–1900), with also the highest IS‐specific ASPR (about 1100 per 100,000; 95% UI: 1000–1200), whereas Sierra Leone led in HS (about 800 per 100,000; 95% UI: 800–900). In contrast, the lowest ASPR was observed in France (SDI 0.88). Overall, ASPRs in 2021 were above the global mean (890.6 per 100,000) in 87 countries and below it in 117 countries and territories.

India also documented the highest number of DALYs associated with pediatric stroke (about 227,300; 95% UI: 159,700–309,000), with HS accounting for about 181,300 DALYs (95% UI: 127,200–245,800). In contrast, Nigeria recorded the highest IS‐related DALYs (about 65,900; 95% UI: 32,500–109,800). China experienced the largest reduction in DALYs over time (EAPC: −5.88; 95% UI: −5.90 to −5.75), whereas Nigeria showed the greatest increase across all stroke types (EAPC of PS: 1.45; 95% UI: 1.17–1.72; EAPC of HS: 0.82; 95% UI: 0.52–1.11; EAPC of IS: 3.52; 95% UI: 3.21–3.83).

Across countries and territories, IS consistently accounted for more prevalent cases than HS (mean: about 7800 vs. about 5500), but HS contributed disproportionately to DALYs (mean: about 9300; 95% UI: 5800–14,300) compared with IS (mean: about 2600; 95% UI: 1600–3900). This pattern underscores the higher long‐term disability burden associated with HS, despite the greater case numbers of IS.

## 5 Discussion

This study provides a comprehensive analysis of the global, regional, and national burden of pediatric stroke from 1990 to 2021, revealing key temporal trends and disparities across age, stroke type, sex, and socioeconomic status. Despite the overall decline in age‐standardized prevalence and DALYs over the past three decades, pediatric stroke remains a major global health concern, particularly in low‐SDI regions and among adolescents [1, 2].

In 2021, we estimated 2.7 million prevalent pediatric stroke cases, representing a modest decline compared with 1990, a trend that reflects improvements in healthcare infrastructure and neonatal care in high‐SDI regions [2, 12]. However, the incidence among adolescents aged 10–19 years increased from about 144,300 cases in 1990 to about 152,500 in 2021, pointing to emerging risk factors, such as trauma, lifestyle behaviors (smoking and alcohol consumption), and rising obesity rates, particularly in high‐SDI settings [3, 5]. HS accounted for 78.3% of DALYs and 85.9% of deaths in 2021, underscoring its disproportionate severity compared with IS. This is consistent with previous findings showing that HS often carries higher mortality and disability due to its catastrophic presentation and limited treatment availability in resource‐constrained environments [6, 9, 13]. Although neurosurgical interventions have improved outcomes in high‐income countries, access to such therapies remains critically limited in LMICs [14, 15].

Age is a crucial determinant of pediatric stroke burden. Neonates (< 28 days) face specific risks, including perinatal complications, intraventricular hemorrhage, and congenital heart disease, whereas adolescents (15–19 years) exhibited the highest HS incidence, reflecting age‐related shifts in risk factor profiles [7, 8]. These findings align with Fullerton et al., who reported higher rates of both IS and HS in older children and adolescents, although population‐level variation persists [13, 16, 17]. Sex‐specific differences were also observed: females demonstrated a higher prevalence of IS, particularly during adolescence, which may relate to hormonal changes, pregnancy, and contraceptive use [18, 19]. Males showed greater susceptibility to HS, potentially linked to higher trauma exposure and the burden of sickle cell disease, which disproportionately affects males in Sub‐Saharan Africa [20, 21]. Tailoring prevention strategies to these demographic differences is essential.

Marked geographic and socioeconomic disparities were evident. Low‐SDI regions, particularly Sub‐Saharan Africa, carried the heaviest burden, with HS prevalence rising from about 146,600 cases in 1990 to about 267,200 in 2021. Contributing factors include limited healthcare infrastructure, shortages of diagnostic and treatment resources, and elevated exposure to infections, malnutrition, and genetic disorders, such as sickle cell disease, which increase pediatric stroke risk by over 200‐fold in African populations [22, 23, 24]. By contrast, high‐SDI regions, notably East Asia, achieved substantial reductions in DALYs, largely due to advances in neonatal care and management of congenital anomalies [25, 26]. This widening disparity highlights the urgent need for investment in pediatric stroke prevention and care infrastructure in LMICs.

Beyond biological factors, environmental and societal determinants contribute significantly. Cold‐related strokes accounted for 5.8% of global stroke DALYs in 2019, with excess mortality linked to moderate cold exposure in regions and countries such as Southern Latin America and India [13, 25, 27]. These findings underscore the importance of climate‐sensitive public health policies. The economic burden is also substantial: in the United States, the mean five‐year cost of pediatric stroke care was estimated at $118,644 per patient, with annual national costs exceeding $42 million [5, 21]. Costs are even more burdensome in LMICs, where families frequently absorb the expenses due to inadequate insurance coverage [12]. Survivors often experience long‐term neurological sequelae, including cognitive impairment, epilepsy, and motor disability, which hinder education and employment opportunities. These consequences reinforce the urgency of expanding prevention, acute management, and rehabilitation efforts.

Based on our findings, three key strategies are recommended [1]: improving access to acute stroke care, particularly for HS in low‐resource settings [2]; strengthening prevention initiatives through control of modifiable risk factors, such as infections, malnutrition, and lifestyle behaviors, with attention to age‐ and sex‐specific patterns; and [3] expanding rehabilitation services, including physical, occupational, and speech therapy, especially in LMICs [5, 28]. Future research should investigate the economic impact of pediatric stroke in resource‐limited regions and assess the effectiveness of emerging interventions such as thrombectomy and telemedicine in improving outcomes [5, 29].

## 6 Strengths and Limitations

This study offers sufficiently detailed assessment to date of the global burden of pediatric stroke, using robust GBD methodology and spanning more than three decades. However, several limitations should be acknowledged. The reliance on modeled estimates may obscure local variability, particularly in regions with poor data availability. It should also be noted that this analysis was not prospectively registered on a public platform, such as the Open Science Framework (OSF), which could potentially introduce reporting biases, although all analyses were conducted following standard epidemiological practices. The ecological nature of the study precludes analysis of individual‐level risk factors. Furthermore, economic and societal impacts, especially in low‐SDI regions, were not fully captured. Despite these limitations, our study provides robust evidence of the ongoing and uneven burden of pediatric stroke and offers valuable insights to inform targeted interventions and guide future research.

## 7 Conclusion

This study provides a comprehensive representation of the global, regional, and national burden of pediatric stroke using modeled estimates from the GBD 2021 study. Our findings demonstrate marked geographical disparities, with the highest age‐standardized rates observed in low‐SDI regions, particularly Sub‐Saharan Africa. Although global trends indicate a decline in age‐standardized incidence, mortality, and DALYs over the past three decades, the absolute number of affected children remains substantial. Adolescents aged 15–19 years and females appear disproportionately impacted, underscoring the importance of age‐ and sex‐specific preventive strategies. HS continues to account for the majority of DALYs and deaths, highlighting the urgent need for targeted management approaches. These results should be interpreted with caution, given the model‐based nature of GBD estimates, but they provide a valuable foundation for guiding health system strengthening, improving access to diagnostics and rehabilitation, and reducing inequities in pediatric stroke care worldwide.

## Author Contributions

Z.Y. and H.D. contributed to methodology design, investigation, and original draft preparation. H.C. and H.H. performed data curation, formal analysis, and visualization. N.W. participated in manuscript review and editing. T.L. and J.T. conceptualized the study, secured funding, validated results, provided resources, supervised the project, and coordinated manuscript revision in response to reviewers' comments. All authors reviewed and approved the final manuscript.

## Funding

This study was funded by the General Project of the Chongqing Natural Science Foundation (Grant cstc2021jcyj msxmX0606) and Key Project of Chongqing Municipal Education Commission (Grant KJ2024004109887015).

## Ethics Statement

The study only used publicly available data and the ethics statement can therefore be waived.

## Consent

The authors have nothing to report.

## Conflicts of Interest

The authors declare no conflicts of interest.

## Supporting information


**Table S1:** Number of Incidence, DALYs and deaths of stroke stratified by stroke type and sex group in pediatric population and estimated annual percent changes from 1990 to 2021.

**Table S2:** Prevalence cases, DALY rates, associated case changes and estimated annual percent change of DALYs in pediatric stroke stratified by global and 21 sociodemographic index (SDI) regions, 1990–2021.

**Table S3:** Prevalence, age‐standardized prevalence rate, DALYs, and estimated annual percent changes of pediatric stroke in the top 5 countries or regions worldwide across stroke type.

## Data Availability

The raw data used in this study were obtained from the Global Burden of Disease (GBD) 2021 dataset, available at http://ghdx.healthdata.org/gbd‐results‐tool. The processed data are provided in the supplementary materials.
